# Clinical model for Hereditary Transthyretin Amyloidosis age of onset prediction

**DOI:** 10.3389/fneur.2023.1216214

**Published:** 2023-07-17

**Authors:** Maria Pedroto, Teresa Coelho, Alípio Jorge, João Mendes-Moreira

**Affiliations:** ^1^Laboratory of Artificial Intelligence and Decision Support (LIAAD), Institute for Systems and Computer Engineering, Technology and Science (INESC TEC), Porto, Portugal; ^2^Department of Computer Science (DCC), Faculty of Sciences (FCUP), University of Porto, Porto, Portugal; ^3^Department of Informatics Engineering (DEI), Faculty of Engineering (FEUP), University of Porto, Porto, Portugal; ^4^Unidade Corino de Andrade, Centro Hospitalar Universitário de Santo António, Porto, Portugal

**Keywords:** ATTRv amyloidosis, feature construction, genealogical features, onset prediction, regression data modeling

## Abstract

**Introduction:**

Hereditary transthyretin amyloidosis (ATTRv amyloidosis) is a rare neurological hereditary disease clinically characterized as severe, progressive, and life-threatening while the age of onset represents the moment in time when the first symptoms are felt. In this study, we present and discuss our results on the study, development, and evaluation of an approach that allows for time-to-event prediction of the age of onset, while focusing on genealogical feature construction.

**Materials and methods:**

This research was triggered by the need to answer the medical problem of when will an asymptomatic ATTRv patient show symptoms of the disease. To do so, we defined and studied the impact of 77 features (ranging from demographic and genealogical to familial disease history) we studied and compared a pool of prediction algorithms, namely, linear regression (LR), elastic net (EN), lasso (LA), ridge (RI), support vector machines (SV), decision tree (DT), random forest (RF), and XGboost (XG), both in a classification as well as a regression setting; we assembled a baseline (BL) which corresponds to the current medical knowledge of the disease; we studied the problem of predicting the age of onset of ATTRv patients; we assessed the viability of predicting age of onset on short term horizons, with a classification framing, on localized sets of patients (currently symptomatic and asymptomatic carriers, with and without genealogical information); and we compared the results with an out-of-bag evaluation set and assembled in a different time-frame than the original data in order to account for data leakage.

**Results:**

Currently, we observe that our approach outperforms the BL model, which follows a set of clinical heuristics and represents current medical practice. Overall, our results show the supremacy of SV and XG for both the prediction tasks although impacted by data characteristics, namely, the existence of missing values, complex data, and small-sized available inputs.

**Discussion:**

With this study, we defined a predictive model approach capable to be well-understood by medical professionals, compared with the current practice, namely, the baseline approach (BL), and successfully showed the improvement achieved to the current medical knowledge.

## 1. Introduction

Hereditary amyloidosis related to variant transthyretin (ATTRv amyloidosis) (1) is a multisystemic disease, with predominant involvement of the heart and the peripheral nervous system. Point mutations in transthyretin, a plasma transport protein, mainly produced in the liver lead to instability of the tetrameric structure of the native protein and disassembly of the monomers that undergo a misfolding process resulting in a widespread deposition of amyloid in the extracellular space of many tissues with consequent organ dysfunction (2). Genetic heterogeneity (more than 130 pathogenic mutations have been described) and phenotypic heterogeneity (variable organ involvement and variable age of onset) are characteristics of this condition, but they are not completely understood. Phenotypic heterogeneity, either the variability of the age of onset or the predominance of a given organ involvement, is driven both by mutation and by genetic background (3). Nonetheless, patients with the same mutation originating in the same region, and even in the same family, may present significant clinical diversity. The variability of the age of onset has been extensively studied and its causes are the object of different types of research, including different strategies to identify genetic modifiers (4, 5).

In recent years, researchers developed several approaches to study and predict future symptom occurrence in patients with different, complex, and irregular diseases. A few examples are breast cancer occurrence and renal artery stenosis (6), Framingham risk functions for cardiovascular diseases (7), and, most recently, genetic frontotemporal dementia (8). In these cases, most of the research focuses on applying statistical-based analysis that is dependent on the dimension of the patient cohorts and sometimes suffers from a lack of enough follow-up temporal data.

In the case of ATTRv amyloidosis, where we have variable penetrance of the gene, the probability of a carrier of a given mutation developing the disease has been extensively studied. This variable penetrance translates into a variable age of onset from the late teens until very old ages and also explains many cases of sporadic presentation of the disease in probands, later shown to have relatives with the same mutation remaining silent throughout their lifespan (9). This is a phenomenon called non-penetrance of the gene. It has been noted in this case that penetrance has been found to be influenced by the mutation, the genetic background, the gender of the patient, and the gender of the transmitting parent (10).

The disease is present worldwide (11), but it was initially described as occurring in clusters or foci related to the same mutation, sometimes known to originate from a common founder, many centuries ago. Most of these clusters are related to the TTRVal30Met mutation (also known as p. TTRVal50Met), but other mutations also show the same behavior (12, 13). The study of large clusters of TTRVal30Met mutation such as those present in Portugal, Sweden, and Japan showed a particular relation between the symptomatic presentation and the early or later age of onset, a positive correlation of the age of onset inside the same family but also an unexpected (14) finding of anticipation of the age of onset, related to the gender of the patient, and the gender of the transmitting parent (15). For subjects belonging to an affected family, the study of the pedigree allows for the definition of the risk of being a carrier of the mutation and to proceed to a blood genetic test (preferably under genetic counseling conditions) to identify those who are carriers of the mutation causing the disease in their relatives (16). However, after this definition, it would be important to predict the age of onset of the disease, the moment when the first symptoms will appear, prompting the need to start one of the presently available disease-modifying treatments, and the moment problems commonly disabling will start impacting their personal, familial, professional, and social life. The development of a sound method to predict the age of onset of a carrier could also contribute to the organization of medical care, defining that the moment carriers should be regularly observed to detect the first symptoms or signs of the disease.

Several studies emphasized the importance of early onset of disease-modifying treatments (17) and we can even consider that pre-symptomatic treatment, when the pathogenic process has started but the functional reserve of affected tissues, such as the heart and the peripheral nerves prevent symptomatic manifestations of this silent evolution would have the best results in the long term. The design of a clinical trial targeting this pre-clinical stage would benefit from the possibility of predicting the age of onset of the asymptomatic carriers based on the knowledge of the age of onset of their relatives. Recently, in Conceição et al. (18), authors discussed the prediction of the age of onset in ATTRv patients and proposed a method currently known as PADO which is presented as based in the clinical practice. Further discussion can be found in Section 4.

We define the age of onset as the moment when a set of characteristic symptoms progressively confluent and more frequent and severe start, disregarding isolated, inconstant, and unspecific complaints. The diagnosis must always be confirmed with the identification of the mutation (when not done previously) and the confirmation of amyloid deposition in any tissue, with the least invasive biopsy, such as abdominal fat, salivary gland, or skin punch biopsies). In the rare cases of recurrent biopsies without evidence of amyloid deposition, the diagnosis is accepted only if objective and unequivocal symptoms of the disease are present and other potential causes have been excluded (19).

Predicting the age of onset for ATTRv amyloidosis patients is difficult as the disease is rare, with genetic and phenotypic heterogeneity and has a wide range of possible age of onset values. Family history can help this evaluation if other family members' ages of onset are known, but we must understand how to valorize this information according to what we know about the variability of the age of onset in many families.

In this study, we follow-up on our previous study on genealogical feature construction (20–22). Our objective is to study, develop, and evaluate an approach to allow time to event the age of onset prediction, in a robust manner, while focusing on genealogical feature construction. This methodology lays the foundation to assess the disease evolution over time.

Our main contributions are (i) the definition of a large set of genealogical-based features constructed from heterogeneous clinical data sets, (ii) the study and comparison of a pool of machine learning algorithms optimized for the age of onset prediction, and (iii) the comparison of the results with an out-of-bag evaluation set, assembled in a different time-frame than the original data.

## 2. Materials and methods

### 2.1. Data

Our data comes from *Unidade Corino de Andrade*, a disease specialized unit officially recognized as a Portuguese National Reference Center. It is integrated into one of the largest Portuguese public hospitals, *Centro Hospitalar Universitário de Santo António*, and its medical professionals treat and maintain accurate patient records since the 1940's. From their records, we started by selecting information from 2,259 patients, diagnosed and followed before 2018. Recently, in order to account and evaluate for possible leakage problems, we added a validation data set and included data from 213 extra patients, diagnosed between 2018 and the end of 2021 (see Table 1). Of note currently, there are 511 patients classified as asymptomatic carriers that are expected to show symptoms in the next few years. Current patients are spread over 932 families, and these numbers tend to increase over time, as new families are diagnosed.

**Table 1:** Two thousand four hundred and seventy-two patients diagnosed with ATTRv amyloidosis in Unidade Corino de Andrade as of December 2021.

**Symptoms**	**Gender**	
Symptomatic (before 2018)	Male	1,258
	Female	1,001
Symptomatic (after 2018)	Male	110
	Female	103
Asymptomatic or pre-symptomatic	Male	178
	Female	333

These results show that there are 511 patients asymptomatic patients as of late 2021.

### 2.2. Baseline model

Currently, for ATTRv amyloidosis, the incorporation of a predicted age of onset to insure a more accurate asymptomatic patient follow-up is not an established practice. One of the most well-known studies that reflect on this problem can be found in Lemos et al. (15).

By taking Lemos et al. (15), we defined a baseline rule model that assumes a linear trend between the age of onset of a patient and that of the transmitting ancestor. In this case, we have


if (father and son) then ŷ=parent(ageonset)-6.06,

if (father and daughter) then ŷ=parent(ageonset)-1.23,

if (mother and son) then ŷ=parent(ageonset)-10.43,

if (mother and daughter) then ŷ=parent(ageonset)-7.43.

To produce comparable results, we added to the baseline a rule for cases where we do not have information regarding the gender or the age of onset of the transmitting ancestor. Therefore, for simplicity purposes, we state that *the patient will most likely feel symptoms of the disease in the 2 years after the prediction time point*. Thus, the last rule states that


if (data unknown) then ŷ=patient(current_age)+2.

These rules, which have different constant values that represent the average difference in the age of onset of all previously diagnosed known pairs of (parent and child), are based on the existence of an anticipation inheritance mechanism. This genetic anticipation, a common factor in most hereditary diseases, indicates that a patient has a higher risk to show symptoms of the disease prior to that of the parent(s). In this case and for each known different pair of (patient, ancestor) gender, we averaged the overall differences of each pair. These values were obtained from the current pool of patients to allow for a sound comparison between the baseline and our machine learning based approach.

We will use this baseline (BL) as a reference in the validation (results in Section 3) of a more powerful predictive approach.

### 2.3. Approach

In this study, we focused on answering two clinical questions formulated in a classification and a regression setting.

In the classification-based setting, our purpose is to infer “*if a patient previously diagnosed with the genetic error will show symptoms of the disease in the next 4 years*.” In this case, we will not be interested in the patient's point age of onset but rather in whether we will be capable of accurately diagnosing them as showing symptoms of the disease. The results will answer how accurate we are capable to diagnose a patient as symptomatic in the period between 2018 and 2021.

In the regression-based setting, our purpose is to infer “when will a patient show symptoms of the disease.” In this study, we are interested in the difference between the ŷ and the *y* values to accurately model this time event.

#### 2.3.1. Patient representation

To answer these problems we start by representing a patient in specific moments in time (age marks). This is not an easy task since we are interested in a formulation that captures the highly variable risk at which a patient will show symptoms of the disease, while focusing on different sets of characteristics, namely, demographic, genealogical, and based on familial disease history. In this case, we chose to construct features that aggregate information from familial connections between patients and their family members. To do this, we implemented a version of the lowest common ancestor algorithm to have a set of valid relations for each patient (23), which helps to supply the aforementioned features. Therefore, overall, for a specific patient and for each age mark, we calculate three sets of features (see Table 2): patient feature set, first-level feature set, and extended feature set. By taking into account these sets of features we characterize our patients in different age marks and later use them to train different sets of models. These are specialized on an age mark and will generate different risks for each patient. In this case, the age marks for which we group our patients are 22, 25, 28, 31, 34, 37, 40, 43, 46, 49, 52, 55, 58, 61, and 64 years old.

**Table 2:** List of 77 original and generated features classified between patient, first level, and extended level.

**Type**	**Description**	**Number of features**
Patient	Patient's gender	1
	Father and mother age onset	1
	Born before father or mother had symptoms	1
	Year of birth, death, and onset	3
	Current age and age of death	2
	Survival current and survival length	2
	Death and onset status	2
	Age of onset (target variable)	1
First level	Number of Children (by gender)	2
	Number of siblings (by gender)	2
	Number of uncles and aunts	2
	Number of nephews and nieces	2
	Number of children with the disease (by gender)	2
	Number of siblings with the disease (by gender)	2
	Number of uncles with the disease (by gender)	2
	Number of nephews with the disease (by gender)	2
	Avg, max and min age of onset of children (by gender)	6
	Avg, max and min age of onset of siblings (by gender)	6
	Avg, max and min age of onset of uncles and aunts	6
	Avg, max and min age of onset of nephews and nieces	6
Extended	Avg, max and min age of onset of patients followed at the clinical unit (by gender)	6
	Avg, max and min age of onset of patients in the family tree (by gender)	6
	Avg, max and min age of onset of early onset patients (patients with age of onset < 50)	6
	Avg, max and min age of onset of late onset patients (patients with age of onset ≥ 50)	6

Overall, we have features that focus on demographic, clinical, and genealogical to familial disease history.

**Figure 1:**
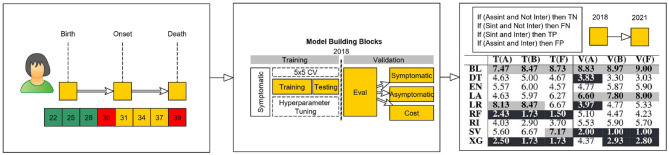
Conceptual model of virtual age scenarios.

The representation of a patient at a specific age mark is only valid if they were still alive both for the classification and regression case and if they were still considered asymptomatic by the medical team in the regression case. As an example, in Figure 1, we show, for a simulated patient, its valid age marks, which range from 22 to 30 years; that the patient is symptomatic, with an age of onset of 30 years; that the asymptomatic ages range from 22 to 28 years old; and that the patient died with 39 years. Therefore, this patient cannot be used in models for ages >39 years (age of death).

#### 2.3.2. Model construction

After the construction of the set of valid age mark instances for each patient, both for the training/ testing and validation sets, we assembled a set of functions to assist the creation of our model construction and evaluation phases (see Figure 1). To evaluate our approach, we selected a temporal validation block (6, 24). To do so, we sliced the data set into two parts: one part containing early treated patients that were used to develop the model, and another part containing the most recently treated patients. We started with a non-random split (6) in which patients that showed symptoms and were diagnosed prior to 2018 were used in the training/ testing sets to check the robustness of the model, while the patients diagnosed after 2018 and the patients that currently have not shown symptoms, but are expected to, were bagged together and used in the validation phase. The definition of training, validation, and testing is done next.

#### 2.3.3. Model evaluation

To compare our approach with the medical baseline, we separated between the patients with known parent(s) age of onset and those without. For that purpose, we developed three types of models: **data set A**, containing patients with previously diagnosed ancestor(s); **data set B**, which contains those for whom we do not have a measurable age of onset for the ancestor; and **data set F**, composed of all available records.

When building a machine learning model, it is essential to evaluate its performance accurately. To do so, each data set (data set A, data set B, and data set F) was further split into three groups namely, training, testing, and validation sets.

In broad terms, the training data set constitutes the reference samples by which an algorithm learns a specific concept. Once the model has been trained, the testing data set is used to assess the performance of the model and to allow for a good tuning of specific hyperparameters capable to balance between over (when the model performs well on the training set but poorly on unseen data) and under fitting (when the model is too simple or has poor performance on both the training and test data). Finally, the validation data set is used to evaluate the model's final performance on unseen data after it has been optimized using the training and testing sets. This set should only be used once, after the model has been optimized, to provide an unbiased estimate of the model's performance on new data and to provide a better indication of how well the model will perform in the real world.

After dealing with the horizontal and vertical break of the data set, i.e., the separation between different sets of features and instances to train/ test and validate our study, we aligned (see Figure 1) each patient's instance according to the age it represented. This alignment allowed for the construction and training of different models according to the age instance of each patient. With this process, each patient has a set of prediction results, which correspond to a risk factor for each age instance. In this case, the prediction of the age of onset of a patient at each age is an independent task which allows for the bagging of sets of patients from different temporal decades. This way, we end up with an independently tuned model for each time horizon and are able to select the best set of hyperparameters and algorithms for relatively small data sets (25).

## 3. Results

In this section, we present the experimental set-up and our results both for the classification as well as regression based approaches.

### 3.1. Experimental setup

As discussed previously, for each asymptomatic patient, we considered the information known at the time at which they were 22, 25, 28, 31, 34, 37, 40, 43, 46, 49, 52, 55, 58, 61, and 64 years old (as long as they were still asymptomatic) and instantiate an individual model for each group of patients. This means that for each age group, and each data set, we have a single experiment with each of the nine different algorithms (i.e., LR, EN, LA, RI, SV, DT, RF, XG, and BL). In total, we ran 405 experiments to compare the results for different patients' ages.

As for our results, these are presented in two settings.

**Figure 2:**
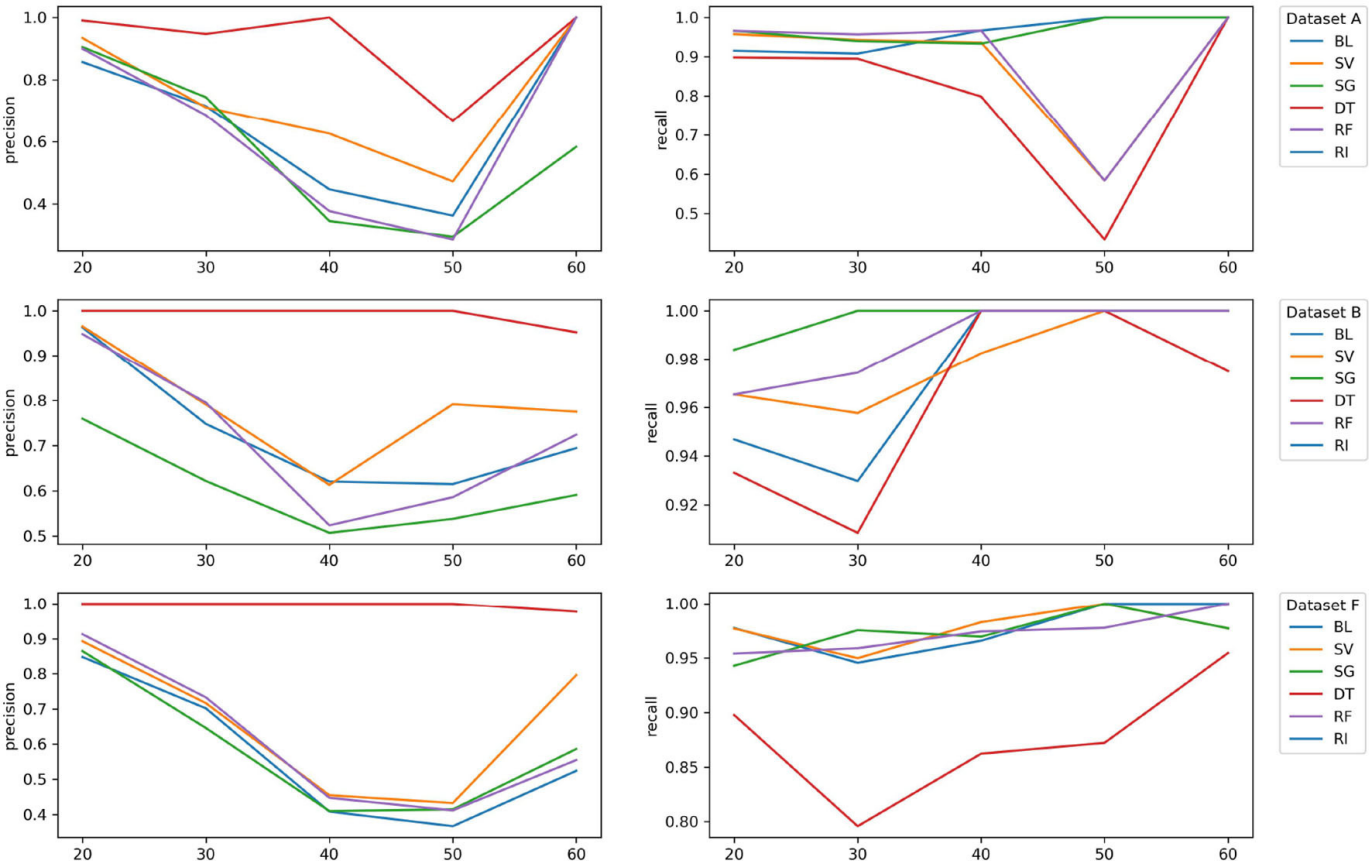
Evolution of precision and recall for V(A), V(B), and V(F) data set (these and other metrics, aggregated in the age dimension, are referenced on Table 3). In the early ages, we are mostly able to identify the patients that will remain asymptomatic. This decreases in the later onset ages (e.g., before 50 years old as opposed to after 50 years old). The best results are given by SV.

Regarding the question if it is viable to check if a patient will feel symptoms of the disease in the next 4 years (classification setting), we present and analyze the validation set both for asymptomatic and symptomatic patients. Therefore, for each patient, we analyzed each prediction age of the last known instance of each patient before 2018 and verify if the calculated age of onset fell in the period set between 2018 and 2021 or not. This resulted in each patient having a single prediction value, which allows us to group all the patients together, independently of the disease stage they were at (see Table 3 and Figure 2). In this case, our metrics of choice will be recall (R) also referenced as true positive rate, which indicates how well a model is capable to identify all symptomatic patients and precision (P), also referred to as positive predictive value, which indicates how precise or accurate a model's symptomatic predictions are.

**Table 3:** Classification-based metrics evaluation.

	**Model**	***avg*(*P*)±*std***	***avg*(*R*)±*std***	***avg*(*F*1)±*std***	***avg*(*A*)±*std***
V(A)	BL	**0.42** **±0.30**	**0.61** **±0.37**	**0.48** **±0.31**	**0.54** **±0.26**
	DT	0.69 ± 0.26	**0.96** **±0.05**	0.77 ± 0.19	0.76 ± 0.20
	EN	0.56 ± 0.31	0.95 ± 0.07	0.66 ± 0.26	0.65 ± 0.24
	LA	0.63 ± 0.33	0.88 ± 0.11	0.68 ± 0.24	0.67 ± 0.24
	LR	0.53 ± 0.33	0.90 ± 0.25	0.62 ± 0.30	0.63 ± 0.25
	RF	0.75 ± 0.30	0.89 ± 0.25	0.80 ± 0.26	0.83 ± 0.18
	RI	0.55 ± 0.31	0.97 ± 0.06	0.65 ± 0.25	0.65 ± 0.23
	SV	**0.92** **±0.25**	0.81 ± 0.26	**0.85** **±0.25**	**0.93** **±0.06**
V(B)	XG	0.66 ± 0.31	0.90 ± 0.25	0.73 ± 0.27	0.77 ± 0.19
	BL	**0.55** **±0.08**	**1.00** **±0.00**	**0.71** **±0.07**	**0.55** **±0.08**
	DT	0.71 ± 0.15	0.98 ± 0.03	0.81 ± 0.10	0.74 ± 0.16
	EN	0.62 ± 0.12	0.99 ± 0.02	0.76 ± 0.09	0.65 ± 0.12
	LA	0.99 ± 0.06	**0.88** **±0.11**	0.92 ± 0.08	0.92 ± 0.08
	LR	0.57 ± 0.10	0.98 ± 0.06	**0.71** **±0.07**	0.57 ± 0.10
	RF	0.77 ± 0.17	0.98 ± 0.03	0.86 ± 0.11	0.80 ± 0.16
	RI	0.59 ± 0.10	1.00 ± 0.01	0.74 ± 0.07	0.62 ± 0.10
	SV	**0.99** **±0.03**	0.97 ± 0.05	**0.98** **±0.03**	**0.97** **±0.03**
V(F)	XG	0.70 ± 0.16	0.99 ± 0.02	0.81 ± 0.11	0.73 ± 0.16
	BL	0.55 ± 0.12	0.91 ± 0.04	**0.67** **±0.09**	0.58 ± 0.08
	DT	**0.55** **±0.18**	**0.98** **±0.03**	0.69 ± 0.14	0.57 ± 0.17
	EN	0.59 ± 0.19	0.95 ± 0.06	0.71 ± 0.12	0.62 ± 0.15
	LA	0.99 ± 0.03	**0.77** **±0.13**	0.86 ± 0.08	0.88 ± 0.08
	LR	0.56 ± 0.21	0.96 ± 0.05	0.68 ± 0.15	**0.56** **±0.19**
	RF	0.65 ± 0.20	**0.98** **±0.03**	0.76 ± 0.14	0.69 ± 0.19
	RI	0.57 ± 0.17	**0.98** **±0.03**	0.70 ± 0.13	0.60 ± 0.16
	SV	**0.99** **±0.02**	0.88 ± 0.08	**0.93** **±0.04**	0.94 ± 0.04
	XG	0.59 ± 0.18	**0.98** **±0.03**	0.72 ± 0.13	0.63 ± 0.16

With a black background, we show the top performers, and in light gray, we show the worst performers. (P, precision; R, recall; A, accuracy; F1, harmonic mean of the precision and recall). Overall, the best results were achieved by SV.

**Figure 3:**
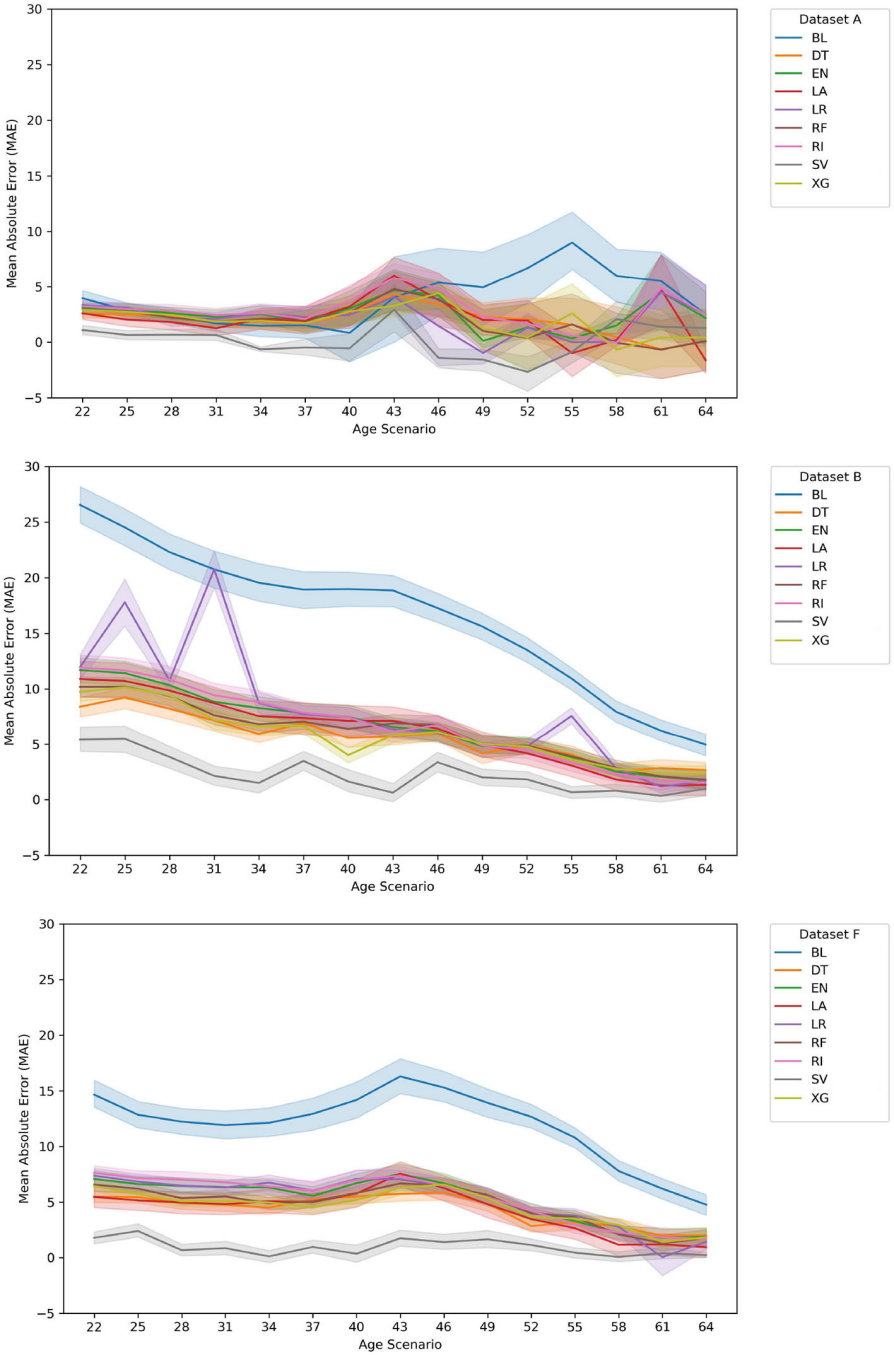
Evolution of MAE for Val(A), Val(B), and Val(F) data sets. For the data set A case, and at the later ages, our approach ranks top when compared with the BL. This also happens in data set B and data set F, notwithstanding the fact that the average error rates of the BL decrease as current age increases. There is some instability in the LR results in the early ages, for data set B resulting of the large number of features. Overall, the SV and XG correspond to the best models for a high majority of age models created.

On the contrary, regarding the prediction of age of onset (regression setting), we analyze the average position (rank) of the mean absolute error (MAE) and the root mean squared error (RMSE) of each pair of (*algorithm* and *data set*) results. We then compare how each model performed over the full set of experiments (see Table 4 and Figure 3). We also analyze and compare the average rank in the training/ testing sets with the one in the validation set. This allows us to infer as to the robustness of our approach and to distinguish our models between **top** and **worst** performers.

**Table 4:** Average MAE ranks of each tuple (algorithm and data set) for model-based imputation with parameter *k* = 10.

	**T(A)**	**T(B)**	**T(F)**	**V(A)**	**V(B)**	**V(F)**
BL	**7.47**	**8.47**	**8.73**	**8.83**	**8.97**	**9.00**
DT	4.63	5.00	4.67	**3.83**	3.30	3.03
EN	5.57	6.00	4.57	4.77	5.87	5.90
LA	4.63	5.97	6.27	**6.60**	**7.80**	**8.00**
LR	**8.13**	**8.47**	6.67	**3.97**	4.77	5.33
RF	**2.43**	**1.73**	**1.50**	5.10	4.47	4.23
RI	4.03	2.90	3.70	5.53	5.90	5.70
SV	5.60	6.67	**7.17**	**2.00**	**1.00**	**1.00**
XG	**2.50**	**1.73**	**1.73**	4.37	**2.93**	**2.80**

T (data set) corresponds to training+testing results. V (data set) corresponds to validation results. With a black background, we have a top (2) **top** performers. With gray coloring, we have a top (2) **worst** performers. Overall, the MAE results are not stable. In the training + testing set, we have as top performers, the RF and XG. In the validation set, we have as top performers SV and XG.

The mean absolute error (MAE) is an evaluation metric used to measure the average absolute difference between the actual and the predicted values in a set of data. It measures how far, on average, the predictions are from the true values, and it is obtained by taking the absolute value of the difference between each actual value and its predicted value and then taking the average of all those absolute differences. As for root mean squared error (RMSE), it corresponds to a metric used to measure the average difference between the actual and predicted values in a set of data. The main difference between MAE and RMSE is that the later (RMSE) is more sensitive to larger differences (outliers) than the first (MAE).

### 3.2. Is it viable to check when will a patient feel symptoms?

For the classification-based evaluation, and by considering the interval between 2018 and 2021, we had to align each patient at the beginning of 2018 and transform the predicted age of onset to a year instance. Due to the lack of a valid prediction model prior to 22 years of age, here we had to exclude nine patients, that are currently asymptomatic.

In the study, we observe that in the early ages, we are mostly able to identify the negative examples (patients that will remain asymptomatic). This result decreases in the later onset ages (e.g., before 50 years old as opposed to after 50 years old). We can see the impact by the peek of the onset age dispersing the results following the period of 30–35 years.

Overall, these results are impacted by the rather small prediction period that ranges between 2018 and 2021. The results of the top and worst performers show that although there is some fluctuation, the overall top performer is the support vector classifier (SV) algorithm for most of the chosen metrics. In the case of recall (R), most algorithms obtain good results, and SV is usually well above 0.9 except in one point for data set A. In fact, when having a closer look at the different ages, it is clear the consistency of the SV algorithm.

### 3.3. When will an asymptomatic patient feel symptoms of the disease?

When comparing the validation results of the MAE error distribution among the different ages (see Figure 3), it is clear that for the data set A case, and at the early ages, our approach ranks the top when compared with the BL although in later years this is not as clear. This fact is likely affected by the decrease in the validation set size throughout the age groups. It is important to reference that this approach suffers from a lack of new asymptomatic patients as the ages increase (presently, the average age of onset of the training/ testing + validation sets is 36.32 years) as well as in the change between the training/testing and validation sets (see Table 1).

As for data set B and data set F, it is clear that our approach shows the best results, with SV being the best-chosen algorithm and XG being the second-best.

Regarding the **average ranks** (see Tables 4, 5), and when comparing the dispersion and difference between the top two performers, we have two main observations. First, on the one hand, the different MAE ranks are not stable between the training/ testing and validation sets. When taking a closer look at the RMSE average ranks, we see that this does not happen. These rank difference between training/testing (T) and validation (V) sets in MAE results suggest that there is a difference in large error distribution in SV results. This can indicate some future problems since we want to have some assurances as to the future performance of the **top** selected approaches.

**Table 5:** Average RMSE ranks of each tuple (algorithm and data set) for model-based imputation with parameter *k* = 10.

	**T(A)**	**T(B)**	**T(F)**	**V(A)**	**V(B)**	**V(F)**
BL	**8.87**	**8.87**	**9.00**	**8.80**	**8.97**	**8.93**
DT	4.87	4.13	4.20	**3.43**	3.63	3.00
EN	5.00	6.00	6.07	4.77	5.80	6.13
LA	5.53	**7.73**	**7.80**	**6.53**	**7.80**	**7.93**
LR	**6.20**	6.33	6.67	4.43	4.90	5.13
RF	4.80	3.13	3.20	4.87	4.10	4.13
RI	4.53	5.53	6.07	5.67	6.07	6.20
SV	**1.27**	**1.07**	**1.00**	**2.33**	**1.07**	**1.00**
XG	**3.93**	**2.20**	**2.00**	4.13	**2.67**	**2.53**

T (data set) corresponds to training + testing results. V (data set) corresponds to validation results. With a black background, we have a top (2) **top** performers. With gray coloring, we have a top (2) **worst** performers. Overall, we have stable rank results since top performers, for the training+testing set, are SV and XG, while in the the validation set, we also have as top performers SV and XG.

Second, that, there is a good degree of stability of the ranks between each vertical data set separation (e.g., between data set A, data set B, and data set F).

## 4. Discussion

This study explores genealogical features in modeling and predicting the age of onset of ATTRv amyloidosis patients. The definition of different sets of data explores different levels of pre-existing information regarding the patients' ancestry.

To compare our approach with current medical practice, we studied works focused on ATTRv amyloidosis guidelines regarding the age of onset of asymptomatic patients, namely, Lemos et al. (15) and Conceição et al. (18).

Our purpose was to provide a concise and clear baseline method, which follows the current medical practice. The baseline needs to be defined in a manner that enables its implementation without major adaptations.

In the first study, authors studied the evolution of the age of onset in a group of families and found the existence of an anticipation mechanism (26), a common factor in hereditary diseases. This fact has been studied for ATTRv amyloidosis in other geographic areas with the studies of Drugge et al. (27), Tashima et al. (28), and Cisneros-Barroso et al. (29). This study, which used a large enough pool set of patient records, presents a statistically based foundation that can foster the development of a wide range of analytical inferences focused on the distribution of the age of onset of ATTRv patients and its genealogical influence.

In a later study (18), a team of doctors with a wide knowledge in treating and following patients diagnosed with ATTRv amyloidosis debated the importance of assessing the predicted age of onset of asymptomatic patients (PADO). As the authors acknowledge, the definition of a prediction value can insure the correct monitoring of known asymptomatic subjects until such time that the onset is medically observed with fewer costs for the public health system. Authors mention that PADO depends on patient mutation, the typical age of onset for that mutation, and the age of onset in family members with ATTRv amyloidosis, specifically its proband (e.g., family first diagnosed case).

When comparing both studies, a few differences emerge. In Lemos et al. (15), researchers study the impact of genealogical inheritance factors and validate their work with data results. In the case of subjects in the asymptomatic status, they only consider information known from their affected relatives and present their results in a clear and concise manner as to allow for the implementation of the resulting rules in a non-biased manner. On the contrary, in Conceição et al. (18), we have a few concerns.

Although we know in different mutations and in many regions, there is a trend in favor of early or late onset with correspondence on the predominant neurological or cardiac phenotype, we also know that there is a major variability in the age of onset and the clinical presentation, even when we consider such subgroups of the disease.

The authors of Conceição et al. (18) indicate that their approach depends on the particular mutation, the typical age of onset for that mutation, and the age of onset in family members with ATTR amyloidosis, without elaborating as to in what manner should the age of onset of family members be used. By this, we mean that authors did not specify if a patient with known family will have a PADO value that gives the average AOO of patients in his/her family tree or will have a PADO that corresponds to the same value, or the result of an anticipation factor when considering the proband and/or immediate family members. These are only a few of the possible considerations that can be made regarding PADO and why it is unclear when it comes to the usage of family data.

We would also like to refer that the authors of Cisneros-Barroso et al. (29), while assessing the hypothesis of an anticipation factor in the AOO in a cohort of Mallorca patients, examine PADO. They state that while in a previous international consensus on ATTRV30M amyloidosis authors recommended to start monitoring asymptomatic carriers 10 years before PADO, their findings suggest that this should be done with caution, specifically when patients come from endemic areas where ATTRv amyloidosis is common.

In summary, when comparing Lemos et al. (15) and Conceição et al. (18), it is clear that while in the first study, authors reflect on the fact that there is an evolution related with the AOO of patients when their family data is taken into consideration, and that despite of the obvious experience of Conceição et al. (18) authors' in treating and following patients, their study lacks a clear mathematical description of the rules that correspond to the V30M variant, which allows for their translation to computational operations. As such, the study of Lemos et al. (15) represents a more suitable baseline (BL) to compare with our study.

Regarding our results for the prediction questions, we have the following issues. Our results in the classification problem show the supremacy of the SV algorithm even though they are impacted by the rather small prediction period. As for the regression problem, our results show the supremacy of the machine learning approaches when compared to the baseline results: in particular, SV and XG are ranked as top performers. In specific, XG results tend to be the most stable, when comparing training/ testing and validation results, namely, with the MAE rank values. Indeed, although SV ranked first in the validation set, its inconsistency between testing and validation sets leads us to rely mainly on XG. In the case of XG, its good position was already expected in part due to the algorithm's strengths and the intrinsic characteristics of the problem of predicting the age of onset of ATTRv amyloidosis patients, with clinical and genealogical data, namely, the existence of missing values, complex data, and small sized available inputs. It is also important to reference that these results might well be influenced by XG built-in capabilities to prevent overfitting while working well with small data, as well as with data with subgroups. The differences in size between the training/ testing and validation set can impact the generalization of each algorithm and can lead to differences between training/ testing and validation results (see Table 1).

Since some of the tried methods showed some instability, namely, in later ages (see Figure 3), from a cautionary perspective, we also suggest the usage of DT, as a more intelligent, but still human perceptible, baseline. In practice, this method represents a simpler approach that, regardless of age group and data set, can function as a computational baseline.

To the best of our knowledge, this is the first work that studies and presents a data-oriented approach that can report the risk of a patient showing symptoms before they appear. We base our results and conclusions on data from a single reference center but with the largest number of patients and families with the same mutation and genetic background known worldwide. The registers we analyzed go back many decades and have been collected and sanitized according to similar criteria. Although this is an important work for guiding the clinical follow-up of Portuguese families, from a cautionary perspective, it would be important to verify its replicability in other disease foci, namely, with different mutations and clinical characteristics such as France, Spain, Sweden, and Japan (30). By this, we mean that even though it may be difficult to apply our conclusions to other centers and groups of patients, we think this study can be a first step in defining the much-needed guidelines for a data-oriented prediction of the age of onset of a carrier of a TTR mutation.

Overall, with this study, we successfully achieved our goals as we defined a predictive model capable to be well-understood by medical professionals since in the top performance, we have tree-based algorithms; compared with the current medical practice, namely, the baseline approach (BL) and successfully showed the improvement with good predictive results. We also defined an adequate experimental approach that enables us to explore different sets of genealogical features and compare their results in a time-to-event prediction setting.

Regarding future work directions, we intend to (i) work on the definition of a modeling strategy to predict the age of onset independently of patients' current age (ensemble guided approach); (ii) work on a modeling strategy that uses genotypic information of the patients, i.e., the genetic mutation of each patient; (iii) study the age limits for which to develop statistical validated models; (iv) attempt to accurately and amply model the costs of asymptomatic patients as well as missed treatments for symptomatic patients; (v) study different approaches to extract valuable information from genealogical data sets; and (vi) explore the definition of another medical baseline that follows the inferences of Conceição et al. (18), although in this case, we would need to integrate information from several different and geographically disperse medical centers. Finally, we add that we believe that there is also a need to focus on studying explainability approaches, such as LIME, in order to understand a few of the irregular trends found in our results and supply medical professionals with a set of workable results for their daily patient assessment.

## Data availability statement

Patient data is not publicly available due to information that could compromise research participant privacy/consent. Requests should be directed to MP, maria.j.pedroto@inesctec.pt or TC, tcoelho@netcabo.pt.

## Ethics statement

The studies involving human participants were reviewed and approved by Centro Hospitalar Universitário de Santo António, Porto, Portugal. The patients/participants provided their written informed consent to participate in this study.

## Author contributions

MP, AJ, and JM-M: conceptualization and methodology. MP: software and data curation. MP, TC, AJ, and JM-M: writing—original draft preparation and writing—review and editing. TC, AJ, and JM-M: supervision and funding acquisition. All authors contributed to the article and approved the submitted version.
